# Objective evaluation of postoperative changes in real-life activity levels in the postoperative course of lumbar spinal surgery using wearable trackers

**DOI:** 10.1186/s12891-020-3102-2

**Published:** 2020-02-04

**Authors:** Masahiro Inoue, Sumihisa Orita, Kazuhide Inage, Miyako Suzuki, Kazuki Fujimoto, Yasuhiro Shiga, Hirohito Kanamoto, Koki Abe, Hideyuki Kinoshita, Masaki Norimoto, Tomotaka Umimura, Takashi Sato, Masashi Sato, Masahiro Suzuki, Keigo Enomoto, Yawara Eguchi, Tsutomu Akazawa, Yasuchika Aoki, Yohei Kawasaki, Seiji Ohtori

**Affiliations:** 1 Department of Orthopaedic Surgery, Eastern Chiba Medical Center, 3-6-2 Okayamadai, Togane City, Chiba 283-8686 Japan; 2 Department of Orthopaedic Surgery, Graduate School of Medicine, Chiba University, 1-8-1 Inohana, Chuo-ku, Chiba City, Chiba 260-8670 Japan; 3 Department of Orthopaedic Surgery, Kohnodai Hospital, National Center for Global Health and Medicine, 1-7-1 Kohnodai, Ichikawa City, Chiba 272-8516 Japan; 4 Department of Orthopaedic surgery, Kanamoto Orthopaedics Clinic, 740-7 Matunaga, Numazu City, Shizuoka 410-0874 Japan; 5 Department of Orthopaedic Surgery, Chiba Cancer Center, 666-2 Nitona-cho, Chuo-ku, Chiba City, Chiba 260-0801 Japan; 6 Department of Orthopaedic Surgery, Center for Orthopaedic science medical innovation Graduated School of Medicine, Chiba University, 1-8-1 Inohana, Chuo-ku, Chiba City, Chiba 260-8670 Japan; 7 Department of Orthopaedic Surgery, St. Marianna University School of Medicine, 2-16-1 Sugao, Miyamae-ku, Kawasaki City, Kanagawa 216-8511 Japan; 8 Biostatistics Section, Clinical Research Center, Chiba University Hospital, 1-8-1 Inohana, Chuo-ku, Chiba City, Chiba 260-8677 Japan

**Keywords:** Lumbar spinal surgery, Amount of activity, Wearable device, Patient-based questionnaire, Objective evaluation

## Abstract

**Background:**

Lumbar spinal disease causes disabilities in performing daily activities. Operative treatments are aimed at pain relief and rapid return to routine activity. Patient-based outcome measures are used to evaluate pathologies and therapeutic effects associated with lumbar spinal disease. Nevertheless, it remains unknown as to how much such treatment improves activity levels.

The purpose of the current study was to measure changes in activity levels before and after lumbar spinal surgery using a wearable activity tracker and to analyze the differences between results and patient-based outcomes.

**Methods:**

Sixty patients who underwent lumbar surgery were studied. The physical activity of participants was objectively evaluated using a wearable Micro-Motion logger system (Actigraph). We measured the amount of activity before and at 1, 3, 6, and 12 months after the surgery to evaluate postoperative changes. The Japanese Orthopaedic Association Back Pain Evaluation Questionnaire, Oswestry Disability Index, Roland-Morris Disability Questionnaire and visual analog scale were used to assess patient-based outcomes of pain and activities of daily living-related scores; we analyzed the relationships between scores and actual activity levels.

**Results:**

The amount of actual activity decreased significantly 1 month after the surgery compared to that during the preoperative period, which then improved after 3 months postoperatively (*p* < 0.01). Furthermore, there was a significant improvement 6 months after the surgery compared to that during the preoperative period (*p* < 0.05). The changes in activity for each period were strongly correlated, regardless of the period. In contrast, a significant improvement was observed at 1 month after the surgery in almost all items of the patient-based questionnaires (*p* < 0.05).

**Conclusions:**

The objective activity tracker demonstrated that lumbar surgery results in the amount of activity decreasing 1 month just after surgery followed by gradual postoperative recovery within 3 months. By contrast, patient-based outcomes showed improvement in 1 month that was significantly different from the change in actual activity, indicating a gap between patient-oriented clinical scores and their actual activities.

## Background

Spinal disorders have a greater burden than most other commonly detected medical conditions. Especially in the elderly population, spinal disorders are more prevalent and may have a more significant impact on health status [1]. Pathological state can sometimes cause disability in daily activities, requiring operative as well as conservative treatment. These treatments are aimed at relief of pain and rapid return to routine activity. Nevertheless, it remains unknown as to how much treatment improves activity levels.

Patient-based outcome measures are used to evaluate pathology and therapeutic effects associated with lumbar spinal disease [2, 3]. While these measurements have the merit of being simple and allow for the evaluation of multiple items, their disadvantage is that objective evaluation can be difficult due to the self-answering design and adventitious missing values in these measurements [4]. Therefore, more objective assessment procedures should be explored and applied to precisely evaluate the postoperative course.

In the field of biophysical monitoring, wearable sensors to capture an individual’s movements and physical activity have been attracting attention with respect to health outcome measurements [5]. These devices objectively collect and store measurements related to the activities of daily living, in addition to exercise, sleeping habits, and vital sign changes. Movement-related acceleration (which in turn correlates with energy consumption) was monitored using accelerometers [6, 7]. In an objective assessment using wristwatch-type wearable trackers, it was reported that patients with rheumatoid arthritis and lower back pain had decreased activity levels compared to healthy participants, and it appeared that the amount of daytime activity reduced due to disease and pain [8, 9]. However, there have been few reports on changes in the amount of actual activity before and after lumbar surgery of patients. Therefore, the current study aimed to evaluate changes in real-life activity levels before and after lumbar spinal surgery using a wearable activity tracker, and to analyzed the differences between the results and patient-based questionnaire outcomes.

## Methods

### Study design

In the current prospective observational study, patients scheduled to undergo surgical treatment for lumbar spinal disease were consecutively recruited at a single institution. The study was approved by the ethics committee of our institution (No.2428). All participants were informed of the purpose of the study, received information, and provided written consent.

### Inclusion and exclusion criteria

The patients were 49 to 88 years old and underwent surgeries at our hospital for lower back pain and lower limb symptoms due to lumbar degenerative disease. The diagnosis of lumbar degenerative disease was made by the treating spine surgeon based on the clinical evaluation, radiographic, and magnetic resonance imaging findings. The surgeries were performed by two spinal surgeons.

In our institution, all patients were required to fill out a medical interview sheet regarding past illness and comorbidities. Patients with illnesses or symptoms that could have affected the amount of activity or questionnaire results were excluded based on the medical interview sheet and physical examinations. Other exclusion criteria were as follows: (1) presence of motor weakness or deficit; (2) presence of severe painful osteoarthropathy; (3) coexisting gait disorder associated with a disease other than degenerative lumbar disease; and (4) psychiatric or cognitive disorders.

### Actual physical activity measurement

The objective physical activity of participants was evaluated using the Actigraph® Micro-Motion logger (Ambulatory Monitors Inc., Ardsley, NY, USA), a wristwatch-shaped waterproof omnidirectional accelerometer (size: 2.5 × 0.9 cm; weight: 14 g), with which acceleration is transduced by a piezoelectric element with a sensitivity of 0.01 G/min, and these voltages were recorded and averaged in 1-min epochs. The Actigraph is an evidence-based tracking system designed for continuous 24-h monitoring and analysis of activity levels and movement counts during both waking and sleeping hours. Each participant wore the logger on the non-dominant wrist for 1 week for each time point, allowing us to calibrate the data for daytime activities (between 8 AM to 6 PM). Data were collected and analyzed using the dedicated Action-W software (version 2.4.15), based on the validated algorithms [10]. Outcome measures included items reported in previous studies for comparisons [7, 10, 11]: the proportional-integrating mode (PIM), the total amount of movement in a 1-min epoch. The PIM provides a high-resolution measurement (range: 0–65,000) of the area under the rectified analog signal, which is designed to quantify more sedentary types of motions. We measured the mean active count (MAC) in PIM as the activity amount before surgery and 1, 3, 6, and 12 months after surgery.

### Patient-based outcome measurements

Clinical symptoms were evaluated using the visual analog scale (VAS) score for lower back pain, leg pain, and leg numbness ranging from 0 (no pain) to 100 mm (extreme pain), the Oswestry Disability Index (ODI) (0–100 points), the Roland-Morris Disability Questionnaire (RDQ) (0–24 points), and the Japanese Orthopaedic Association Back Pain Evaluation Questionnaire (JOABPEQ). JOABPEQ, RDQ, and ODI are all Japanese questionnaires, and their validation with the original language has been validated [12, 13]. The first two items, VAS and ODI, can be used to evaluate lumbar pain, leg pain, and numbness; however, these do not directly measure symptoms that occur in association with certain postures and activities. The RDQ was designed specifically to measure the impact of lumbar pain on the quality of life. The JOABPEQ includes 25 questions based on the RDQs and Short Form 36 (SF-36). Scores were calculated based on the answers to questions in five domains: pain-related disorders, lumbar spine dysfunction, gait disturbance, social life dysfunction, and psychological disorders. The score for each domain was calculated according to official guidelines and ranged from 0 to 100 points with lower scores indicating greater symptom severity, which is deemed proportional to the patient’s clinical condition [14, 15].

### Statistical analysis

To evaluate the change in the activity amount, the postoperative MAC divided by the preoperative MAC multiplied by 100 was calculated as the percentage change in each period. First, the percentage change before and after the surgery was calculated with a linear mixed model for repeated measures. Additionally, to evaluate the relationship between changes in the amount of activity for each period, a single regression analysis was performed using the former as the explanatory variable and the latter as the objective variable in the two periods.

Next, to evaluate the transition of patient-based outcome as well as activity level, the relationship between the preoperative and postoperative score was evaluated in each item using the Wilcoxon signed-rank test.

All data are reported as means ± standard deviations, unless otherwise indicated. We defined the significance level at 5% and used SAS Version 9.4 for all statistical analyses in the current study.

## Results

The current study included 60 patients (30 men and 30 women). The mean age was 70.7 ± 8.4 years. The mean BMI was 24.5 ± 4.4 kg/m^2^. The mean duration of morbidity was 50.9 ± 49.9 (min 2, max 180) months. The disease breakdown included 42 cases of lumbar spinal stenosis, 13 cases of lumbar spondylolisthesis, and five cases of degenerative lumbar scoliosis. The surgical procedure included 26 cases of decompression surgery and 34 cases of fusion surgery (Table 1).

**Table 1:** The demographic data of the patients

No. of patients	60
Age, mean (range), yr	70.7 ± 8.4 (49–88)
Sex (Male / Female)	30/ 30
Body mass index (kg/m^2^)	24.5 ± 4.4
Duration of illness, (range), month	50.9 ± 49.9 (2–180)
Diagnosis	
Lumbar spinal stenosis	42 (70%)
Lumbar spondylolisthesis	13 (22%)
Degenerative lumbar scoliosis	5 (8%)
Surgical method	
Decompression	26 (43%)
Fixation	34 (57%)

### Comparison of the activity amounts before and after the surgery

**Fig. 1:**
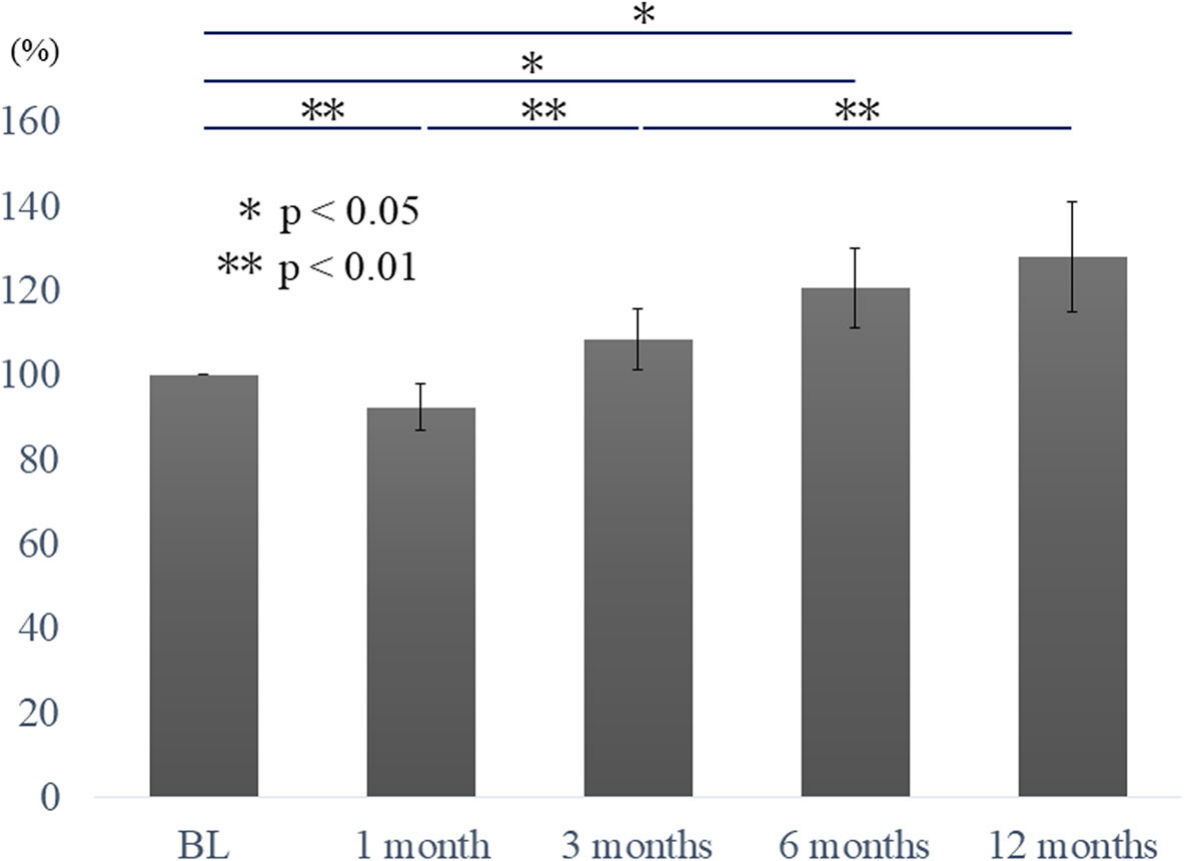
Percentage change before and after surgery. Activity amount was significantly lower at 1 month after the surgery compared to the other time points. In addition, at 6 and 12 months postoperatively, the amount of actual activity improved significantly compared to the baseline. BL, baseline

Some data were not available for each period due to wearable device failures (1 month, *n* = 3; 3 months, *n* = 1; 6 months, *n* = 2; 12 months, n = 1), incomplete questionnaires (1 month, *n* = 7; 3 months, *n* = 6; 6 months, *n* = 8; 12 months, n = 6), and missed patient visits (1 month, *n* = 7; 3 months, *n* = 6; 6 months, *n* = 4; 12 months, *n* = 9). The percentage change of the mean active counts compared to the baseline in each period were 92.3 ± 5.6 at 1 month postoperatively, 108.2 ± 7.2 at 3 months postoperatively, 120.5 ± 9.4 at 6 months postoperatively, and 127.9 ± 13.2 at 12 months postoperatively (Table 2). The MAC was significantly lower at 1 month after the surgery than during the preoperative period and it improved after 3 months postoperatively. At 12 months postoperatively, there was a significant increase even compared to that at 3 months (*p* < 0.01). Compared to the preoperative period, the MAC increased significantly at 6 and 12 months postoperatively (*p* < 0.05) (Fig. 1). Regarding the relationship of change in the MAC in each period, correlations were observed in all periods in a single regression analysis. Among them, a strong correlation was found between the preoperative and other periods (*p* < 0.01), and the strongest correlation was between 1 month postoperatively and 3 months postoperatively (*r*^2^ = 0.792, *p* < 0.001) (Table 3).

**Table 2:** Percentage change was calculated by dividing the mean active count for each period by the mean active count at baseline. The value from the analysis was calculated using repeated measurement for percentage change. SD, standard deviation; SE, standard error

	n	Mean active count, SD	Percentage change from baseline, SD	Repeated measurement, SE
Baseline	60	3474 ± 1698	100	100
1 month	43	3279 ± 1673	90.9 ± 31.8	92.3 ± 5.6
3 months	47	3547 ± 1700	104.2 ± 42.2	108.2 ± 7.2
6 months	46	3777 ± 2017	111.6 ± 49.7	120.5 ± 9.4
12 months	44	3912 ± 1879	125.6 ± 104.4	127.9 ± 13.2

**Table 3:** Correlation of mean active count at baseline and 1, 3, 6, and 12 months after surgery for each patient

		Mean active counts	
		*R*^2^	*p* value
Baseline -	1 month	0.5113	<0.001
	3 months	0.5766	<0.001
	6 months	0.5712	<0.001
	12 months	0.4527	<0.001
1 month -	3 months	0.7920	<0.001
	6 months	0.2911	<0.001
	12 months	0.4479	<0.001
3 months -	6 months	0.4149	<0.001
	12 months	0.5288	<0.001
6 months -	12 months	0.5869	<0.001

### Comparison of the patient-based questionnaire before and after the surgery

For the patient-based questionnaires, the scores were significantly improved in most of the periods compared to those during the preoperative period. For JOABPEQ, pain-related disorder significantly improved (56.5 ± 40.5 at 1 month, 71 ± 34.9 at 3 months) postoperatively compared to that during the preoperative period (34.3 ± 30.2; *p* < 0.05). There was little change after 3 months. This was similar to gait disturbance and social life dysfunction. Psychological dysfunction was significantly improved at 1 month after the surgery and there was no significant change after that. In contrast, lumbar spine dysfunction showed improvement of score 3 months after the surgery. As for ODI, RDQ, and VAS, we found a significant improvement in the scores at 1 month after the surgery (*p* < 0.01). The ODI and RDQ scores further improved at 12 months, while lower back pain and lower limb numbness showed a slight increase with the VAS (Table 4).

**Table 4:** Scores of the patient-based questionnaires before and 1, 3, 6, and 12 months following surgery. Abbreviations: JOABPEQ, Japanese Orthopaedic Association Back Pain Evaluation Questionnaire; ODI, Oswestry Disability Index; RDQ, Roland-Morris Disability Questionnaire; VAS, visual analog scale

	Baseline	1 month	3 months	6 months	12 months
JOABPEQ					
Pain-related disorder	34.3 ± 30.2	56.5 ± 40.5*	71.0 ± 34.9*	61.6 ± 36.8*	67.7 ± 36.0*
Lumbar spine dysfunction	46.8 ± 29.7	50.7 ± 29.4	58.3 ± 28.6*	55.9 ± 32.5*	63.4 ± 30.9*
Gait disturbance	20.5 ± 20.3	42.1 ± 27.1*	46.4 ± 30.2*	45.9 ± 33.0*	45.9 ± 30.2*
Social life dysfunction	34.5 ± 17.8	44.9 ± 22.0*	46.2 ± 20.4*	45.1 ± 23.8*	49.8 ± 22.9*
Phycological disorder	38.0 ± 19.6	48.2 ± 17.6*	48.3 ± 15.9*	45.6 ± 18.2*	47.0 ± 14.7*
ODI	51.6 ± 16.2	35.8 ± 17.0*	36.4 ± 19.4*	37.0 ± 20.8*	31.4 ± 19.5*
RDQ	13.8 ± 4.6	9.8 ± 5.7*	9.1 ± 7.1*	9.5 ± 7.0*	8.1 ± 5.9*
VAS					
Low back pain	7.3 ± 2.4	3.7 ± 3.3*	2.9 ± 3.0*	3.0 ± 2.7*	3.7 ± 3.2*
Lower limb pain	7.4 ± 2.6	3.5 ± 3.4*	3.1 ± 3.0*	3.2 ± 3.3*	3.2 ± 3.4*
Lower limb numbness	7.2 ± 2.6	3.2 ± 3.1*	3.6 ± 3.4*	3.8 ± 3.4*	4.5 ± 3.4*

* *p* < 0.05 as compared with baseline

## Discussion

We measured actual changes in activity amount before and after lumbar surgery using wearable activity trackers The amount of activity decreased 1 month after surgery, followed by gradual recovery within 3 months after the surgery, and there was a significant improvement 6 months after the surgery. Moreover, the actual change in activity amount for each period was strongly correlated regardless of the period. by contrast, for patient-based questionnaires, improvement was significant 1 month after the surgery in almost all items, and thereafter change was not significant.

There are several reports on the change in postoperative physical activity using accelerometers. For joint surgery, Bolink et al. reported that the physical performance improved 1 year after total knee arthroplasty compared to that before surgery using an inertial measurement unit [16]. By contrast, Smuck reported that, in 28 cases of lumbar spinal canal stenosis, the amount of activity remained unchanged between before surgery and 6 months postoperatively [17]. In our study, the actual amount of activity showed a temporal decrease 1 month after surgery, while it gradually recovered 3 months after surgery. Furthermore, the measured actual activity significantly improved 6 months after surgery compared to the preoperative period. We believe that the amount of activity decreased with pain and physical fitness due to surgical invasion at the point of 1 month after surgery, and it increased with improvement of pain and symptoms after 3 months. The transition of the activity amount of each patient strongly correlated in any period regardless of the preoperative symptom and surgical procedure. Because the change in activity from 1 to 3 months after surgery was very strongly correlated, the reduction of activity amount due to surgical invasion in the early postoperative period and the subsequent improvement showed similar trends regardless of the patient. This implies that the amount of postoperative activity might be predicted from the amount of preoperative activity.

Regarding the trend of subjective patient-based outcomes, improved scores were observed at 1 month after the surgery for all items. Most items after 3 months postoperatively became constant, and the scores were not improved. This result was similar to that of a previous report [18], but deviated from our data in terms of the actual change in physical activity measured by the wearable activity tracker. There are some reports that low back pain affects the amount of activity [19, 20]; however, especially VAS, that reflects clinical symptoms, improved on the early postoperative days, and it was very different from the change of amount of activity. This result was different from that of previous reports that found that low back pain affects activity. This suggests that pain and clinical symptoms may contribute to a decrease in the activity level, but that the postoperative improvement of symptoms is not directly linked to improvement in activity amount.

The advantage of our study is that we obtained frequent objective data using wearable terminals from short to medium-term and showed a more detailed postoperative course.

Moreover, we showed that there was a dissociation between the actual perioperative change of the amount of activity and the change of subjective evaluation using traditional measurements. This should be considered in the clinical situation to build more effective treatments for lower back pain patients.

There were some limitations to our study. First, because the diseases and surgical procedures of patients participating in the study were different, this influence could not be excluded. Nevertheless, we believe that the change in activity amount before and after surgery was strongly correlated even if the diseases and surgical procedures were different, and this suggests that a similar recovery process follows for lumbar spinal surgery in general. We plan to further investigate the change in activity amount for each disease and surgical procedure. Next, we could not evaluate changes in patient satisfaction, medical cost, and related factors as an influence on activity improvement; these will be evaluated in the future. Furthermore, the sample size was small, and we were not able to consider age and sex separately for analysis. Therefore, it is necessary to involve larger groups in the future.

In the current study, the objective evaluation using a wearable activity tracker was useful in evaluating the actual activities of the postoperative patients, and made it possible to evaluate the actual quality of life before and after surgery, which is difficult to assess using only the traditional subjective patient-based outcomes.

## Conclusions

The objective activity tracker demonstrated that in lumbar surgery, the amount of activity decreased 1 month after the surgery with a gradual recovery within 3 months after the surgery. Moreover, the change in activity amount in each period was strongly correlated irrespective of the period, and the preoperative activity amount had a large influence on postoperative activity amount. By contrast, subjective patient-based outcomes showed improvement in 1 month, different from the change in actual activity, suggesting that the traditional evaluation lacks the ability to express nuances in actual activities in postoperative patients.

## Data Availability

The datasets used and/or analysed during the current study are available from the corresponding author on reasonable request.

## Abbreviations

ADL: Activities of daily living
BL: Baseline
JOABPEQ: Japanese Orthopaedic Association of Back Pain Evaluation Questionnaire
MAC: Mean active count
ODI: Oswestry Disability Index
PIM: Proportional-integrating mode
RDQ: Roland- Morris Disability Questionnaire
VAS: Visual analog scale
